# Practical and Operative Gynecology

**Published:** 1897-06

**Authors:** 


**Practical and Operative Gynecology.** By J. Clarence Webster, M.D. Pp. xiv., 295. Edinburgh: Young J. Pentland. 1896.

This is a useful little book for practitioner and student. It is divided into three parts: Case-taking (including physical examination), minor therapeutic measures, and operative measures. The various directions are given in a very clear and concise manner, and operative details are fairly complete. We do not think that "half a syringeful" (p. 139) is a sufficiently exact dosage for the injection of cocaine or any other drug; and on p. 155 the sterilised salt solution of 6 per cent. should read .6 per cent. It would be better if the figures were referred to in the text itself, instead of leaving the reader to find out the connection between the illustration and the text.

